# Exploring causality in the association between circulating 25-hydroxyvitamin D and colorectal cancer risk: a large Mendelian randomisation study

**DOI:** 10.1186/s12916-018-1119-2

**Published:** 2018-08-14

**Authors:** Yazhou He, Maria Timofeeva, Susan M. Farrington, Peter Vaughan-Shaw, Victoria Svinti, Marion Walker, Lina Zgaga, Xiangrui Meng, Xue Li, Athina Spiliopoulou, Xia Jiang, Elina Hyppönen, Peter Kraft, Douglas P. Kiel, The SUNLIGHT consortium, Xia Jiang, Elina Hyppönen, Peter Kraft, Douglas P. Kiel, Caroline Hayward, Archie Campbell, David Porteous, Katarina Vucic, Iva Kirac, Masa Filipovic, Sarah E. Harris, Ian J. Deary, Richard Houlston, Ian P. Tomlinson, Harry Campbell, Evropi Theodoratou, Malcolm G. Dunlop

**Affiliations:** 1 Colon Cancer Genetics Group, Medical Research Council Human Genetics Unit, Medical Research Council Institute of Genetics & Molecular Medicine, Western General Hospital, The University of Edinburgh, Edinburgh, EH4 2XU UK; 2 West China School of Medicine/West China Hospital, Sichuan University, Chengdu, 610041 People’s Republic of China; 3 Centre for Global Health Research, Usher Institute of Population Health Sciences and Informatics, The University of Edinburgh, Edinburgh, EH8 9AG UK; 4 Department of Public Health and Primary Care, Institute of Population Health, Trinity College Dublin, University of Dublin, Dublin 24, D02 PN40, Ireland; 5 Program in Genetic Epidemiology and Statistical Genetics. Department of Epidemiology, Harvard T.H.Chan School of Public Health, 677 Huntington Avenue, Boston, MA 02115 USA; 6 Unit of Cardiovascular Epidemiology, Institute of Environmental Medicine, Karolinska Institutet, Nobels vagen 13, Stockholm, 17177 Sweden; 7 Australian Centre for Precision Health, University of South Australia Cancer Research Institute, University of South Australia, Adelaide, SA 5001 Australia; 8 Population, Policy and Practice, University College London, Great Ormond Street, Institute of Child Health, WC1E 6BT, London, UK; 9 Institute for Aging Research, Hebrew SeniorLife, 1200 Centre Street, Boston, MA 02131 USA; 10 Department of Medicine, Beth Israel Deaconess Medical Center and Harvard Medical School, Boston, MA 02115 USA; 11 Broad Institute of Harvard and Massachusetts Institute of Technology, Boston, MA 02142 USA; 12 MRC Human Genetics Unit, MRC Institute of Genetics & Molecular Medicine, The University of Edinburgh, Western General Hospital, Edinburgh, EH4 2XU UK; 13 Generation Scotland, Institute of Genetics and Molecular Medicine, The University of Edinburgh, Western General Hospital Edinburgh, Crewe Road, Edinburgh, EH4 2XU UK; 14 Agency for Medicinal Products and Medical Devices, Department for Quality, Safety and Efficacy Assessment, Zagreb, Croatia; 15 Department of Surgical Oncology, University Hospital for Tumours, Sestre milosrdnice University Hospital Centre, Zagreb, Croatia; 16 School of Medicine, University of Zagreb, Zagreb, Croatia; 17 Centre for Cognitive Ageing and Cognitive Epidemiology, The University of Edinburgh, Edinburgh, UK; 18 Centre for Genomic and Experimental Medicine, Institute of Genetics and Molecular Medicine, The University of Edinburgh, Edinburgh, UK; 19 Department of Psychology, The University of Edinburgh, Edinburgh, UK; 20 Division of Genetics and Epidemiology, The Institute of Cancer Research, Sutton, Surrey, SM2 5NG UK; 21 Institute of Cancer and Genomic Sciences, University of Birmingham, Birmingham, UK

**Keywords:** Vitamin D, Colorectal cancer, Mendelian randomisation

## Abstract

**Background:**

Whilst observational studies establish that lower plasma 25-hydroxyvitamin D (25-OHD) levels are associated with higher risk of colorectal cancer (CRC), establishing causality has proven challenging. Since vitamin D is modifiable, these observations have substantial clinical and public health implications. Indeed, many health agencies already recommend supplemental vitamin D. Here, we explore causality in a large Mendelian randomisation (MR) study using an improved genetic instrument for circulating 25-OHD.

**Methods:**

We developed a weighted genetic score for circulating 25-OHD using six genetic variants that we recently reported to be associated with circulating 25-OHD in a large genome-wide association study (GWAS) meta-analysis. Using this score as instrumental variable in MR analyses, we sought to determine whether circulating 25-OHD is causally linked with CRC risk. We conducted MR analysis using individual-level data from 10,725 CRC cases and 30,794 controls (Scotland, UK Biobank and Croatia). We then applied estimates from meta-analysis of 11 GWAS of CRC risk (18,967 cases; 48,168 controls) in a summary statistics MR approach.

**Results:**

The new genetic score for 25-OHD was strongly associated with measured plasma 25-OHD levels in 2821 healthy Scottish controls (*P* = 1.47 × 10^− 11^), improving upon previous genetic instruments (F-statistic 46.0 vs. 13.0). However, individual-level MR revealed no association between 25-OHD score and CRC risk (OR 1.03/unit log-transformed circulating 25-OHD, 95% CI 0.51–2.07, *P* = 0.93). Similarly, we found no evidence for a causal relationship between 25-OHD and CRC risk using summary statistics MR analysis (OR 0.91, 95% CI 0.69–1.19, *P* = 0.48).

**Conclusions:**

Despite the scale of this study and employing an improved score capturing more of the genetic contribution to circulating 25-OHD, we found no evidence for a causal relationship between circulating 25-OHD and CRC risk. Although the magnitude of effect for vitamin D suggested by observational studies can confidently be excluded, smaller effects sizes and non-linear relationships remain plausible. Circulating vitamin D may be a CRC biomarker, but a causal effect on CRC risk remains unproven.

**Electronic supplementary material:**

The online version of this article (10.1186/s12916-018-1119-2) contains supplementary material, which is available to authorized users.

## Background

Colorectal cancer (CRC) is the third most commonly diagnosed cancer worldwide and is one of the leading causes of cancer-specific death [1]. A variety of risk factors have been identified, including low 25-hydroxyvitamin D (25-OHD) [2]. 1,25 dihydroxyvitamin D3 or calcitriol, the active metabolite of 25-OHD, binds to the nuclear vitamin D receptor and subsequently takes effect by maintaining cellular homeostasis and controlling cell growth [3, 4]. Postulated mechanisms for the apparent protective effect of 25-OHD include effects on transcriptional regulation of anticancer target genes involved in proliferation, apoptosis, differentiation, inflammation, invasion and metastasis [4]. Meta-analysis of prospective observational studies involving more than one million participants provided evidence of an inverse association between a 10 ng/mL increment in circulating 25-OHD level and a 26% decreased CRC risk [5, 6]. Given the high prevalence of vitamin D deficiency worldwide [7], especially for high latitude areas such as Scotland [8], and the fact that deficiency can be rectified by dietary supplementation, there is compelling rationale to investigate the contribution of 25-OHD to CRC incidence in the general population.

The associations between vitamin D and CRC reported in observational studies could be biased by reverse causality or confounding factors. Potential confounding factors include body mass index (BMI) [9], diet low in vitamin D, or amount of time spent outdoors [10], each of which may separately influence CRC risk. These could potentially compromise true benefits of any interventions on circulating 25-OHD level. Although the effect of modifying 25-OHD levels can be verified by traditional randomised controlled trials of vitamin D supplementation, these would be prohibitively costly and lengthy in duration. The “VITamin D and OmegA-3 TriaL (VITAL)” was launched in 2010 to investigate the effect of vitamin D supplementation on cancer and cardiovascular disease outcomes [11]. Although 20,000 participants will be recruited to the trial, it could still be underpowered to detect the potential effect on a single type of cancer given the relatively low frequency of CRC occurrence.

**Fig. 1:**
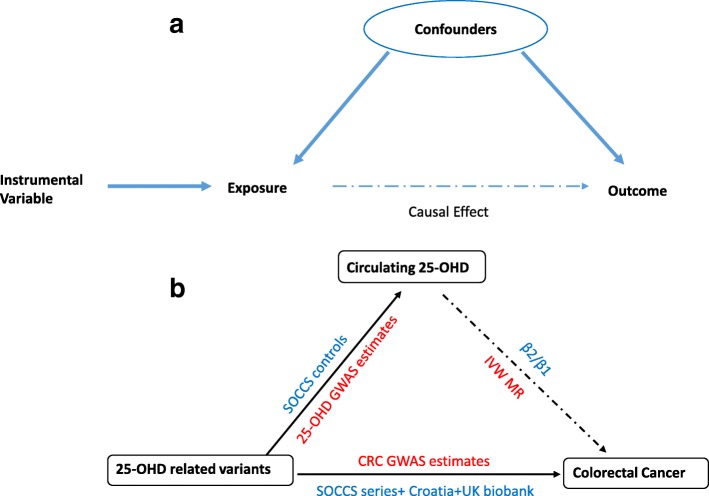
**a** Conceptional framework of Mendelian randomisation (MR). The instrumental variable is based on genome-wide significant single nucleotide polymorphisms from independent studies of the association between the exposure of interest (serum 25-hydroxyvitamin D (25-OHD) concentrations) and the outcome (colorectal cancer (CRC)). The effect of an instrumental variable should be independent from the confounding factors and should affect CRC risk only through exposure. In the presence of a causal relationship, the association between instrumental variable and CRC would be expected to be proportionate to its association with the serum 25-OHD concentrations, given the relationship between the serum 25-OHD concentrations and CRC risk. Figure adapted from Timpson et al. [65]. **b** Basic design of our MR on the causal effect of 25-OHD on CRC risk. The blue text of outer contour showed individual level MR analysis. β1 is the regression coefficient of instrumental variable (IV) on exposure (25-OHD level) using controls from the Scotland Colorectal Cancer Study (SOCCS); β2 is the regression coefficient of IV on outcome (CRC) using SOCCS series, Croatia and UK biobank case control studies. Causal effect is estimated by the ratio of β2 and β1. The red text of inner contour showed summary statistics MR analysis. Effect sizes of IVs on 25-OHD and CRC are extracted from two GWAS meta-analyses and causal estimate is derived from an inverse variance-weighted MR analysis

Mendelian randomisation (MR) is one of the emerging approaches to strengthen causal inference based on the instrumental variable (IV) method [12]. The conceptual framework of MR is shown in Fig. 1a. A typical MR study uses genetic variants as the IV, assuming that risk alleles for a certain phenotype are randomly allocated during gamete formation [13]. There are some basic assumptions for a valid IV in MR studies [14]. The first is the relevance assumption, which means that instrumental genetic variants should be significantly associated with the exposure; the second assumption requires no association between the IV and confounders of the exposure–outcome relationship. The third is the exclusion restriction assumption, indicating that these variants should affect the outcome solely through the exposure. If the MR assumptions are satisfied then the potential causal effect can be inferred based on the observed IV–exposure and IV–outcome associations. Published MR studies so far have not found support a causal relationship between 25-OHD and CRC [15–17]. Our group previously performed two MR studies to investigate the possible causal effects of plasma 25-OHD on CRC risk. We did not detect a significant effect of 25-OHD on CRC risk using the conventional MR approach [15]. However, analysis of Bayesian predictor scores across various hypotheses prioritised causal models accounting for hidden pleiotropy and confounding over the reverse causality hypothesis [18]. The implemented methodology accounted for confounding by unknown factors and allowed pleiotropic relationships; hence, the results are not dependent on strong and often unrealistic assumptions of the classical MR methods.

It is worth noting that, in all previous MR studies, only four genetic variants (rs2282679, rs12785878, rs6013897, rs10741657) [19] were used to build the instrument. Recently, with the sample size of genome-wide association studies (GWAS) accumulating rapidly, two further genetic loci associated with circulating 25-OHD levels were identified (rs10745742 and rs8018720) [20]. Simulation studies found that incorporating more genetic variants into a single instrument by computing genetic risk scores (GRS) could improve the instrument strength and accuracy of estimation [21, 22], highlighting the necessity to re-evaluate the causal effect of 25-OHD on CRC.

Therefore, we designed this MR study to obtain causal estimates of the association between 25-OHD and CRC (Fig. 1b). Six genetic variants associated with 25-OHD level were used as the IV. MR analysis was performed using both individual level data and two-sample summary statistics.

## Methods

### Individual level MR

#### Studies

Five CRC case–control studies from Scotland, UK and Croatia totalling 10,725 CRC cases and 30,794 controls were included in the individual level MR (Additional file 1: Table S1). The Scottish case–control CRC series consisted of three studies of a total of 6278 cases and 14,692 controls, including (1) 1012 cases and 1012 controls from Scotland 1 (COGS study) [23, 24]; (2) 494 cases from the Study of Colorectal Cancer in Scotland (SOCCS) [25] and 1522 population-based controls without prior history of malignant tumours from the Lothian Birth Cohorts (LBC) 1921 and 1936 [26]; and (3) 4772 cases and 2221 population-based controls from SOCCS [25] and additional 9937 population controls without prior history of CRC from the Generation Scotland-Scottish Family Health Study (GS:SFHS) [27, 28]. The fourth study included 3683 cases and 15,642 controls matched by age, sex, date of blood draw, ethnicity and region of residence from the UK biobank cohort [29]. Finally, a case–control CRC study from Croatia consisting of 764 cases and 460 population-based controls was also included in the analysis. Details of study genotyping, quality control procedures and imputations are presented in Additional file 1 and elsewhere [30, 31]. A total of 9940 cases and 22,848 controls with genotyping data were included after extensive quality control procedures (Additional file 1). Each study was approved by the respective institutional ethics review board and performed in accordance with the Declaration of Helsinki.

#### Genetic variants as 25-OHD instruments

We created an IV for 25-OHD using four genetic variants previously shown to be associated with *25-OHD* (rs3755967, rs10741657, rs12785878, rs17216707) [19] and two new single nucleotide polymorphisms (rs10745742, rs8018720) identified by our recent SUNLIGHT Consortium GWAS meta-analysis [20]. This meta-analysis of GWAS of serum 25-OHD concentrations included data from SOCCS. To obtain an unbiased IV that could be applied in our study population, a meta-analysis of 29 cohorts including 77,354 individuals of European ancestry was re-run, excluding the SOCCS samples. Summary statistics (including beta estimates for alleles increasing circulating 25-OHD level, standard error and *P* value) of the genetic variants on 25-OHD were extracted afterwards.

#### Statistical analysis

We created a weighted GRS for each individual in SOCCS/GS, UK biobank and Croatia datasets using the six 25-OHD-associated candidate variants. These variants were weighted by effect sizes of 25-OHD increasing alleles from the SUNLIGHT GWAS meta-analysis excluding SOCCS samples. Unweighted GRS was also generated based on the counts of alleles associated with increased level of 25-OHD for each participant.

First, we tested the association between the 25-OHD GRS and log-transformed 25-OHD levels (nmol/L) in a sub-set of SOCCS controls (*n* = 2821) by applying a univariable linear regression model. We also calculated the F-statistic to evaluate the strength of the genetic instrument, and an F-statistic < 10 was considered as a weak instrument effect [21]. Second, we examined the association between our instrumental GRS of 25-OHD and common confounders including age, sex, BMI, physical activity, assessment centre, smoking status and alcohol consumption based on available data in SOCCS (*n* = 9746) and UK biobank (*n* = 11,382) controls to test the potential violation of the second MR assumption. We also searched the NHGRI-EBI GWAS Catalogue (https://www.ebi.ac.uk/gwas/ accessed in February 2018) to identify any reported associations between the six variants and potential confounders. If the second MR assumption was violated in one of the studies, we performed sensitivity analysis by excluding the corresponding study. We also applied multivariable linear regression models adjusting for age, sex and BMI to obtain the IV–exposure association estimates based on availability of each dataset. Next, the association between GRS and CRC risk was assessed by a logistic regression model in the three Scottish case–control series (Scotland1, SOCCS/GS, SOCCS/LBC), Croatia and UK biobank datasets, adjusting for age, sex and BMI (based on data availability). Using the coefficient ratio method proposed by Wald [32], we measured the causal effect by calculating the ratio of the IV regression coefficient from the IV–outcome association analysis and the IV regression coefficient from the IV–exposure association, and then estimated the standard error based on the Taylor expansion [33, 34].

Estimates from these five datasets were combined by using the inverse variance meta-analysis under a random effects model. The observed *P* value < 0.10 for the χ^2^ Q test indicated no significant heterogeneity among included datasets. Considering potential diverse aetiology of tumours in different anatomical locations, we also performed stratified MR analyses in patients with tumours in proximal, distal colon and rectum using available individual-level data.

### Summary statistics MR

#### Studies

We investigated the relationship between the IV for 25-OHD and CRC using summary data from six previously reported GWAS of CRC [30, 31]. Briefly, these GWAS included individuals of European ancestry from the following studies: CCFR1, CCFR2, COIN, FINLAND, UK1 and VQ58 [35–37] (details in Additional file 1: Table S1). Together with the Scottish case–control series, Croatia and UK Biobank studies we included 18,967 cases and 48,168 controls across 11 individual GWASs (Additional file 1: Table S1). Comprehensive details on the cases and controls are available in previously published work [30, 31, 35–37]. After standard quality control procedures, 17,716 cases and 40,095 control individuals were included in the analysis. All studies were approved by their respective institutional review boards and conducted with appropriate ethical criteria in each country and in accordance with the Declaration of Helsinki.

#### Statistical analysis

Effects of the six genetic variants on 25-OHD (25-OHD increasing alleles) were extracted from the SUNLIGHT GWAS meta-analysis and effects of these variants on CRC risk were extracted from the CRC GWAS meta-analysis results of 11 case–control studies (Additional file 1: Table S1, Table S6). We also checked if any of the known CRC risk variants were in linkage disequilibrium (r^2^ > 0.01) with the 25-OHD associated variants in the CRC GWAS meta-analysis results. We applied a range of MR methods using summary genetics data, namely an inverse variance-weighted (IVW) average of associations for IVs [38], and a median-based method [39]. Egger MR [40] was conducted to explore the potential bias introduced by pleiotropy.

IVW MR combines causal effects of candidate variants estimated following the IVW method as proposed by Burgess et al. [38]. As shown by the formula below, X_k_ refers to the effect size of variant k on the exposure, Y_k_ represents the effect size of the same variant on the outcome, and σ_Yk_ is the standard error of Y_k_. In addition, to evaluate potential heterogeneity among causal effects of different variants, the χ^2^ Q test was employed, and a *P* value of less than 0.10 was regarded as significant heterogeneity.

$$ \widehat{\beta}=\frac{\sum_{k=1}^K{X}_k{Y}_k{\sigma}_{yk}^{-2}}{\sum_{k=1}^K{X}_k^2{\sigma}_{yk}^{-2}} $$

$$ Se\left(\widehat{\beta}\right)=\sqrt{\frac{1}{\sum_{k=1}^K{X}_k^2{\sigma}_{yk}^{-2}}} $$

Considering that unmeasured pleiotropy could lead to violation of the exclusion restriction assumption and bias the MR findings, we employed the MR-Egger regression method that aims to identify and adjust for unbalanced pleiotropy. Additionally, the MR-Egger approach can provide unbiased and minimally biased estimates even in the presence of no causal association and substantial directional pleiotropy [40]. A significant difference of an intercept from zero (*P* < 0.05) suggests existence of unbalanced pleiotropy.

To further evaluate the robustness of possible causal effect when some of the genetic variants in the analysis are not valid IVs and IV assumptions are violated, we also employed median-based methods to derive the causal estimates [39]. As a sensitivity analysis, causal estimates from IVW and MR-Egger were calculated using robust regression in addition to standard linear regression, and penalization of weights of each variant was also applied for IVW, MR-Egger and median-based estimates [41]. A *P* value of less than 0.05 was considered as statistically significant for causal estimates for our MR. In addition, given these six variants are located in multiple genes with diverse function, which could introduce potential pleiotropy, we also conducted a sensitivity analysis with different combinations of variants, starting with rs10741657 plus rs12785878 (in *CYP2R1* and *DHCR7* genes affecting 25-OHD synthesis) and sequentially adding rs17216707, rs10745742, rs8018720 and rs3755967.

### Power estimation

We estimated the power of our study according to the method provided by Brion et al. [42]. The six 25-OHD-related variants explained approximately 2.84% of 25-OHD variation [20]. We fixed the type I error as α < 0.05 and employed a range of effect estimates from odds ratio (OR) 0.6 to 0.98 per standard deviation increased 25-OHD level. Assuming true causal effect of vitamin D is similar to the effect observed in the SOCCS study (OR 0.83 per standard deviation of increased circulating 25-OHD) we would have a power 0.72 for the individual level approach using 9940 CRC cases and 22,848 controls from the UK biobank, Croatia and Scottish CRC case–control series. The study had sufficient power (80%) to detect the causal effects of a 19% or larger decrease in CRC risk per standard deviation increase of 25-OHD. The power for the summary level approach reached 0.80 for a causal effect larger than 14.3% decreased CRC risk per standard deviation increase of 25-OHD. Power estimation for a range of causal effects as well as proportions of 25-OHD variation explained by the six genetic variants is summarised in Additional file 1: Table S3.

All statistical analyses were performed using PLINK 1.90 and R (version 3.3.0) package ‘MendelianRandomization’ [43].

## Results

We tested the MR assumptions using SOCCS and UK biobank individual level data. The MR relevance assumption was tested in SOCCS controls (*n* = 2821) with available circulating 25-OHD levels. Both weighted and unweighted GRS were significantly associated with the log-transformed 25-OHD levels in a univariable linear regression model (weighted GRS: *P* = 1.47 × 10^− 11^, unweighted GRS: *P* = 8.47 × 10^− 9^) and after adjustment for age, sex and BMI (weighted GRS: *P* = 1.37 × 10^− 11^, unweighted GRS: *P* = 5.72 × 10^− 10^). We calculated the F-statistic to evaluate the strength of the genetic instrument [21]. The linear regression showed an F-statistic of 46.0 for weighted GRS and 33.7 for unweighted GRS, suggesting the absence of a weak instrument effect (F > 10). The association between the instrument and possible confounders was tested in SOCCS and UK biobank controls. The genetic instrument of six variants on 25-OHD was not significantly associated with any of the common cofounders including age, sex, height, weight, BMI, physical activity, smoking status, alcohol consumption and assessment centre (*P* > 0.05, Additional file 1: Table S2). By searching the GWAS catalogue, we identified no significant association between any of the six variants and common confounders either. None of the known CRC variants were in linkage disequilibrium (r^2^ > 0.01) with the six 25-OHD variants.

No direct association was observed between the weighted or unweighted GRS and CRC risk in SOCCS, Croatia or UK biobank datasets (Table 1). Detailed results of individual level MR analysis for each dataset are summarised in Table 2. Both univariable and multivariable models adjusted for age, sex and BMI, when appropriate, showed no causal effects of 25-OHD on CRC risk in Scotland 1, SOCCS/GS, SOCCS/LBC, Croatia and UK biobank case–control studies. Overall, the result of individual level MR analysis under a multivariable model suggested no significant causal effect of 25-OHD concentration on CRC risk using the weighted GRS (OR 1.03 per unit increased log-transformed 25-OHD, 95% C 0.51–2.07, *P* = 0.931). No significant heterogeneity was observed among each dataset (*P*_het_ = 0.227). Similarly, we did not find a statistically significant causal effect when an unweighted GRS was employed as the IV (OR 1.12, 95% CI 0.51–2.45, *P* = 0.785). The results of stratified analysis did not support a significant causal effect of 25-OHD on risk for proximal, distal or rectal tumours (detailed results in Additional file 1: Table S5).

**Table 1:** Two-stage regression coefficients for Mendelian randomisation analysis using genetic risk score in individual level data

	GRS-25OHD coefficient (95% CI)^b^	GRS-CRC coefficient (95% CI)^c^				
	SOCCS controls	Scotland 1	SOCCS/GS	Croatia	SOCCS/LBC	UK biobank
Univariable model						
Weighted score	0.055 (0.039 to 0.071)	0.036 (−0.055 to 0.128)	− 0.009 (− 0.045 to 0.026)	0.072 (− 0.048 to 0.191)	− 0.029 (− 0.133 to 0.076)	− 0.022 (− 0.061 to 0.017)
Unweighted score	0.046 (0.031 to 0.062)	0.037 (−0.055 to 0.128)	− 0.005 (− 0.041 to 0.030)	0.110 (− 0.010 to 0.230)	0.001 (− 0.104 to 0.106)	− 0.008 (− 0.047 to 0.031)
Multivariable model^a^						
Weighted score	0.057 (0.040 to 0.073)	0.109 (−0.011 to 0.230)	− 0.026 (− 0.081 to 0.029)	0.074 (− 0.047 to 0.194)	− 0.031 (− 0.136 to 0.074)	− 0.024 (− 0.047 to 0.031)
Unweighted score	0.051 (0035 to 0.068)	0.080 (−0.038 to 0.197)	− 0.022 (− 0.077 to 0.032)	0.114 (− 0.007 to 0.235)	− 0.003 (− 0.108 to 0.102)	− 0.011 (− 0.050 to 0.029)

^a^Multivariable regression model adjusted by age, sex and BMI for Scotland 1, SOCCS/GS and UK biobank, age and sex for Croatia, sex for LBC.MD

^b^Change in log-transformed 25-OHD (nmol/L) per unit increase in GRS

^c^Change in logit CRC risk per unit increase in GRS

*25-OHD* 25-hydroxyvitamin D, *CI* confidence interval, *CRC* colorectal cancer, *GRS* genetic risk score, *LBC* Lothian Birth Cohort, *SOCCS* Scotland Colorectal Cancer Study

**Table 2:** Main results of Mendelian randomisation analysis using individual level data

Cases/controls	Causal estimate (odds ratio)^b^ (95% CI)					Overall estimate^c^	*P* value	*P*_het_^d^
	Scotland 1	SOCCS/GS	Croatia	SOCCS/LBC	UK biobank			
	932/942	4551/8804	689/441	461/1444	3301/11382			
Univariable model								
Weighted score	1.92 (0.36–10.27)	0.84 (0.44–1.62)	3.69 (0.41–33.34)	0.59 (0.09–3.99)	0.67 (0.33–1.38)	0.85 (0.55–1.33)	0.481	0.531
Unweighted score	2.20 (0.30–16.19)	0.89 (0.41–1.93)	10.71 (0.71–161.41)	1.02 (0.11–9.84)	0.85 (0.36–1.96)	1.03 (0.61–1.73)	0.920	0.440
Multivariable model^a^								
Weighted score	6.85 (0.77–60.81)	0.63 (0.24–1.67)	3.82 (0.41–35.23)	0.57 (0.08–3.87)	0.87 (0.44–1.73)	1.03 (0.51–2.07)	0.931	0.227
Unweighted score	4.70 (0.46–48.41)	0.65 (0.22–1.88)	11.21 (0.76–164.33)	0.93 (0.10–8.66)	0.81 (0.38–1.75)	1.12 (0.51–2.45)	0.785	0.222

^a^Multivariable regression model adjusted by age, sex and BMI for Scotland 1, SOCCS/GS and UK biobank, age and sex for Croatia, sex for LBC.MD

^b^Change in CRC risk per unit log-transformed 25-OHD (nmol/L)

^c^Overall estimates were obtained by meta-analyses under random-effect model

^d^*P*_het_, *P* values of χ^2^ Q test for heterogeneity

*25-OHD* 25-hydroxyvitamin D, *CI* confidence interval, *CRC* colorectal cancer, *GRS* genetic risk score, *LBC* Lothian Birth Cohort, *SOCCS* Scotland Colorectal Cancer Study

**Fig. 2:**
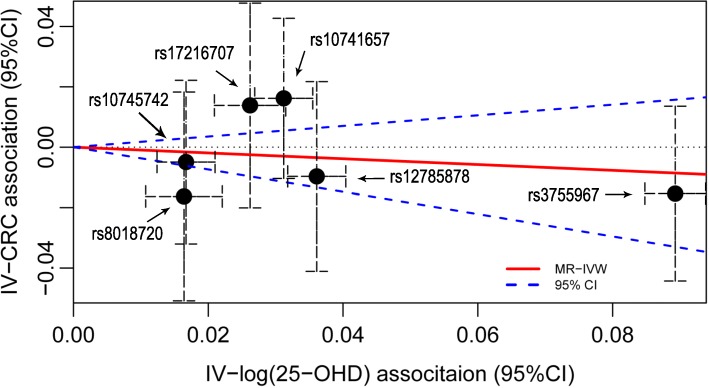
Association of 25-hydroxyvitamin D (25-OHD) affecting genetic variants with log transformed 25-OHD concentration and colorectal cancer risk. The slope of the red line is the causal estimate derived from inverse variance-weighted (IVW) Mendelian randomisation and slope of the blue dash line represents the 95% confidence interval of IVW estimate

As shown in Fig. 2, for the summary statistics IVW MR, no statistically significant causal effect of 25-OHD on CRC risk was identified either (OR 0.91 per unit increased log-transformed 25-OHD, 95% CI 0.69–1.19, *P* = 0.475). MR-Egger regression did not identify evidence of significant horizontal pleiotropy (*P* = 0.657) and the MR-Egger analysis did not observe any statistically significant causal effect (OR 0.83, 95% CI 0.51–1.34, *P* = 0.452). In addition, no significant heterogeneity was detected among the causal estimates of the six variants (*P*_het_ = 0.547). Effects of each single variant on both 25-OHD and CRC are presented in Table 3. Estimates derived from the median-based methods did not show a statistically significant causal effect (simple median method: OR 0.80, 95% CI 0.49–1.30, *P* = 0.375). Detailed results using standard linear regression, robust regression and penalisation are summarised in Table 4. Sensitivity analysis using different combinations of variants did not identify any significant causal effects either (detailed results presented in Additional file 1: Table S4).

**Table 3:** Summary of genetic variants used as instrumental variables in summary statistics approach

ID	Gene	Effect allele	Chromosome	Beta (25-OHD)	Se (25-OHD)	Beta (CRC)	Se (CRC)
rs10741657	CYP2R1	A	11	0.0312	0.0022	0.0162	0.0135
rs10745742	AMDHD1	T	12	0.0167	0.0022	−0.0049	0.0138
rs12785878	NADSYN1/ DHCR7	T	11	0.0361	0.0022	−0.0097	0.016
rs17216707	CYP24A1	T	20	0.0262	0.0027	0.0138	0.0173
rs3755967	GC	C	4	0.0893	0.0023	−0.0154	0.0148
rs8018720	SEC23A	G	14	0.0164	0.0029	−0.0163	0.0176

*Beta* regression coefficients of GWAS meta-analysis, *se* standard error, *CRC* colorectal cancer

**Table 4:** Main results of Mendelian randomisation analysis in 18,967 colorectal cancer cases and 48,168 controls using summary statistic approach

Methods	Causal estimates (95% CI)^a^	*P* value	*P*_int_
IVW	0.91 (0.69–1.19)	0.475	NA
Robust IVW	0.90 (0.74–1.09)	0.318	NA
Penalised IVW	0.91 (0.69–1.19)	0.475	NA
MR-Egger	0.83 (0.51–1.34)	0.452	0.657
Robust MR-Egger	0.83 (0.67–1.03)	0.09	0.61
Penalised MR-Egger	0.83 (0.51–1.34)	0.452	0.657
Simple median	0.80 (0.49–1.30)	0.375	NA
Weighted median	0.84 (0.62–1.15)	0.278	NA
Penalised weighted median	0.84 (0.62–1.15)	0.278	NA

^a^Change in colorectal cancer risk per unit log-transformed 25-hydroxyvitamin D (nmol/L)

*CI* confidence interval, *IVW* inverse variance weighted, *MR* Mendelian randomisation, *NA* not available, *P*_*int*_ *P* value of Egger regression test on the intercept

## Discussion

In the largest MR study to date, we employed a new IV comprising a genetic score that captures more of the genetic contribution to circulating 25-OHD than has ever been possible before, linked to a large meta-analysis of GWAS for CRC risk in well-matched European populations with similar ambient exposure to vitamin D-making UVB sunlight. We aimed to determine whether the relationship between 25-OHD and CRC risk was causal. We employed several MR methods, including individual level MR analysis, summary level IVW, Egger MR and median-based MR. We used six genetic variants (rs3755967, rs12785878, rs17216707, rs10741657, rs10745742, rs8018720) associated with 25-OHD serum levels as IVs [20]. However, none of the implemented approaches supported a causal association between lower plasma 25-OHD and elevated CRC risk.

Previous retrospective and prospective observational studies establish beyond all reasonable doubt that there is an association between lower circulating 25-OHD levels and elevated CRC risk [5, 6]. The issue is whether this is a causal relationship. However, randomised controlled trials have failed to demonstrate beneficial effects of vitamin D supplementation on CRC or colorectal adenoma recurrence as an intermediate endpoint. For instance, the Women’s Health Initiative trial did not show any effects of 1000 mg of elemental calcium and 400 IU of vitamin D_3_ supplementation on CRC incidence among postmenopausal women [44]. Similarly, daily supplementation with vitamin D_3_ (1000 IU), calcium (1200 mg) or both after removal of colorectal adenomas did not reduce the risk of recurrent colorectal adenomas [45]. Albeit questioning the potential causal role of 25-OHD in the development of CRC, these trials are widely criticised for short follow-up or lacking proof for effective 25-OHD modification (due to low dose of supplementation) [46–48]. More recently, in human studies, it has been shown that functional genetic variants in the vitamin D receptor may also influence any protective response to vitamin D in preventing adenomas, which merits further stratified investigation of the possible effect [49]. Similarly, experimental studies using rodent models of colon cancer treated with high dietary vitamin D were inconsistent in their conclusions. In particular, a causal relationship between high dietary vitamin D and low colon cancer risk was supported by studies using a mouse model of bacteria-driven colitis and colon cancer [50], and in mice fed with new Western-style diet [51], but not in a rat model of familiar colon cancer [52].

A randomised trial in average risk populations of sufficient size and duration to establish definitively whether or not vitamin D supplementation prevents CRC as the primary endpoint seems unlikely to ever be feasible. Hence, MR methods offer an alternative approach that might provide clarity on whether 25-OHD is causally associated with CRC risk. There is a pressing need for designing and investing in future trials on the effects of vitamin D in high-risk population subgroups.

Our previous MR study did not detect a statistically significant causal effect of 25-OHD on CRC [15]. Another recent MR study with 11,488 CRC cases did not show a causal relationship between circulating vitamin D level and CRC risk [17]. However, the conclusions might have been limited by lower statistical power. Insufficient power has been a major shortcoming of MR studies, because genetic variants usually explain only a very small proportion of the exposure variation on the liability scale. Those four variants could only explain 3.6% to 5.2% [53, 54] of variance on 25-OHD, thus leading to potentially low statistical power. Our previous study included 2001 CRC cases and 2237 controls, but only reached a power of 0.35 to detect 25% decreased risk per standard deviation increase in 25-OHD [15]. We recently reported the largest ever GWAS on circulating 25-OHD concentrations in which we identified two additional genetic loci contributing to the genetic architecture of 25-OHD [20]. Using these six variants, we developed a stronger instrument compared with the previous four-variant instrument (F-statistic 46.0 vs. 13.0 in SOCCS controls) [15]. However, the overall heritability calculated using linkage disequilibrium score regression analysis [20] was modest, with 2.84% out of 7.5% overall heritability explained by the identified GWAS variants. Although the addition of new GWAS variants provided only limited improvement in the strength of the IV, overall statistical power was substantially improved in our current 25-OHD–CRC MR analysis. With data from the largest GWAS studies on 25-OHD and CRC, as well as more individual CRC cases involved in this MR study, we have a power of 0.80 at the α level of 0.05 to identify a 19% decreased CRC risk per standard deviation increase in 25-OHD for the individual level approach using 9940 CRC cases and 22,848 controls from the UK biobank CRC case–control dataset, Croatia and Scottish CRC case–control series, and a power of 0.80 to identify a 14.3% decreased CRC risk for the summary level approaches using 17,716 cases and 40,095 controls across 11 individual GWASs.

The validity of MR estimates of causal effects requires that several assumptions be held. First, for the relevance assumption, we only included the strongest independent variants identified by the largest GWAS so they were all robustly associated with the exposure. Second, none of the genetic variants used in our analysis were cited by the NHGRI-EBI Catalogue of published GWAS as associated with known CRC risk confounders (such as height, BMI, alcohol consumption, smoking, type II diabetes, inflammatory bowel disease, adenomas) [55]. Furthermore, our genetic instrument was not associated with age, sex, BMI, smoking status, alcohol consumption, physical activity and assessment centre, suggesting no effects of violated IV second assumption due to tested confounders on final study conclusion. However, we cannot rule out the possibility of association between our IV and an unknown and/or unmeasured confounding factor. Finally, to assess violations of the exclusion restriction assumption or ‘no pleiotropy’, we employed a range of methods known to robustly account for horizontal pleiotropy, including MR-Egger and a weighted median approach. All of the methods showed similar results and MR-Egger intercept indicated no evidence of pleiotropic effects, suggesting robust null findings.

Our study had sufficient power and an appropriate design to formally address the hypothesis of a causal relationship between low circulating vitamin D and CRC risk. We also used a range of various MR approaches. Another strength of our study was the availability of collected information on known confounding factors such as height, weight, BMI, age and sex, which allowed testing the MR assumptions of independent associations between IV and confounders. However, there were some limitations too. Firstly, due to the low proportion of 25-OHD variance (2.84%) explained by the genetic variants and relatively small sample size, our individual level data analysis did not reach the desired power (< 0.80) assuming true causal effects of 25OHD on CRC risk was similar to the effect observed in the observational SOCCS case–control study (OR 0.83). The study had sufficient power to identify a causal effect larger than 21.7% decreased CRC risk per 25-OHD standard deviation. Although the summary statistics approach included a larger sample size, we only had a power of 0.49 if the true causal effect was less than 10% decreased CRC risk per 25-OHD standard deviation. Similarly, both approaches were underpowered if the real proportion of 25-OHD variance explained by the IV was 2% and below. Secondly, for individual level analysis, circulating 25-OHD levels from the SOCCS dataset were measured in the Scottish population, which manifested a significantly lower average level compared with other European populations [8]; this could possibly weaken the strength of our genetic instrument. A weak IV is an issue for the summary two-step MR approach too. The MR estimates are known to be biased towards the null in the presence of a weak IV (F statistics < 5) [56, 57]. This is similar to regression dilution bias in an observational study due to non-differential measurement errors. However, given the strength of the IV (F-statistic 46.0) used in the present analysis and the large sample size in the summary level approach, the bias towards the null is unlikely to affect our results. We also cannot exclude the possibility of collider bias due to the non-representative selection of participants into the study cohorts. Selection bias is present to some degree in all epidemiological studies. Evidence of a ‘healthy volunteer’ selection bias has been described for the UK biobank [58, 59]. The collider bias can lead to an association between the IV and the outcome in the absence of a causal effect as well as to underestimation of real causal effects in some cases [60]. It seems, though, that in most cases collider bias effect is smaller than pleiotropy or population stratification bias [60]. Finally, as in many previous MR studies, the current paper is based on the assumption of a linear effect between CRC risk and 25-OHD levels. Indeed, two recent studies on CRC have shown a linear relationship between 25-OHD and CRC [61, 62]. In particular, results from a recent dose–response meta-analysis of observational studies [61] as well as the analysis of the EPIC study [62] support a linear relationship between 25-OHD and CRC. Nevertheless, it is still possible that the assumption of linearity may not hold true. There are some recently suggested IV methods that can test non-linear exposure–outcome effects, but the methods are not fully developed yet [63, 64]. Furthermore, these approaches require access to individual level data, which is a limiting factor for many MR studies including ours. Finally, although application of a linear IV in the case of a non-linear relationship between the exposure and outcome could not give any insight into the shape of the relationship, it is still possible to provide population-averaged causal effects [64].

## Conclusions

In conclusion, this MR study provides further evidence that genetically determined lower circulating levels of 25-OHD are unlikely to have a causal effect on CRC risk with strength on the order of the effects previously reported in observational studies. Observed associations may be due to confounders and reverse causation, although a very small causal effect of 25-OHD on CRC risk cannot be ruled out. Future research might be best focused on understanding the mechanisms of the relationship between CRC and circulating 25-OHD.

## Additional file

Additional file 1: Study description, imputation and genetic analysis and supplementary **Tables S1-S6.** (DOC 211 kb)

## Abbreviations

25-OHD: 25-hydroxyvitamin D
BMI: body mass index
CI: confidence interval
CRC: colorectal cancer
GRS: genetic risk scores
GWAS: genome-wide association study
IV: instrumental variable
IVW: inverse variance weighted
LBC: Lothian Birth Cohort
MR: Mendelian randomisation
OR: odds ratio
SOCCS: Study of Colorectal Cancer in Scotland
