# Supplementary material for: Exploring causality in the association between circulating 25-hydroxyvitamin D and colorectal cancer risk: a large Mendelian randomisation study
Source: BMC Med. 2018 Aug 14;16:142. doi: 10.1186/s12916-018-1119-2 (PMC6090711; doi:10.1186/s12916-018-1119-2)
Supplement: Supplementary file 1 — Study description, imputation and genetic analysis and supplementary Tables S1-S6. (DOC 211 kb) [file 12916_2018_1119_MOESM1_ESM.doc]

**Additional file**

**Studies for Individual level MR**

***Scottish case-control CRC series***

Scottish case-control CRC series consist of a total of 6,278 cases and 14,692 controls including: a) 1,012 cases and 1,012 controls from Scotland 1 (COGS study);b) 494 cases from the Study of Colorectal Cancer in Scotland (SOCCS) and 1,522 population-based controls without prior history of malignant tumours from the Lothian Birth Cohorts (LBC) 1921 and 1936; c) 4,772 cases and 2,221 population based controls from SOCCS and additional 9,937 population controls without prior history of colorectal cancer from Generation Scotland-Scottish Family Health Study (GS:SFHS). Genotyping of SOCCS and GS samples was conducted by OmniExpressExome BeadChip 8v1.1, 8v1.250, 8v1.3 or 8v1.4 (Illumina Inc., San Diego, CA). COGS samples were genotyped using Illumina HumanHap300 and HumanHap240S arrays. LBC cohorts were genotyped using the Illumina610-Quadv1 chip. Ethics approval for the COGS study was obtained from the multicentre research ethics committee. The SOCCS study was approved by the MultiCentre Research Ethics Committee for Scotland (REC reference number: 01/0/05) and by the Research and Development Office of NHS Lothian (reference number: 2003/W/GEN/05). GS:SFHS received ethics approval from the NHS Tayside Committee on Medical Research Ethics (REC Reference Number: 05/S1401/89).

A total of 5,950 CRC cases and 11,025 controls with genotype data were included after extensive quality control procedures. Individuals were excluded with: high missing rate (>0.05%, n=162), history of cancer for control individuals including non-melanoma cancers (n=932) and other benign conditions including adenomas, appendix etc (n=64), discordant sex information (n = 64), individuals recruited twice and first-degree relatedness (n = 2,550), evidence of non-white European ancestry using Principal Component Analysis (PCA) in conjunction with 1000G samples (n = 89; cut-off based on visual assessment of the top two principal components of the controls), abnormal heterozygosity (>3 standard deviations from mean, n=47) and sampling issues (n=84).

Plasma 25-OHD was measured in a subset of 2,821 control and 1,598 cases from SOCCS by liquid chromatography-tandem mass spectrometry (LC-MS/MS) method. We used SOCCS controls to measure association between plasma 25-OHD and IV. In order to balance the effect of different seasons when blood samples were taken, a May-standardisation algorithm was applied on the 25-OHD level for each participants as previously described.

***Case-control study from UK biobank***

UK biobank is a large cohort study approved by North West Multicentre Research Ethics Committee (Reference 11/NW/0382) and with more than 500,000 individuals recruited. Biological samples of these participants were genotyped using the custom-designed Affymetrix UK BiLEVE Axiom array on an initial 50,000 participants and Affymetrix UK Biobank Axiom® array on the remaining 450,000 participants. Genotyping was done at the Affymetrix Research Services Laboratory in Santa Clara, California, USA, from Affymetrix. The two arrays had over 95% common content. The procedure of genotyping and quality control is presented in detail at <https://biobank-ctsu-ox-ac-uk.ezproxy.is.ed.ac.uk/crystal/docs/genotyping_qc.pdf> and elsewhere

A UK biobank CRC case-control study included 3,683 cases and 15,642 population based control individuals. Individuals with ICD9 codes 153.0, 153.1, 153.2, 153.3, 153.4, 153.6, 153.7, 153.8, 153.9, 154.0, 154.1 and ICD10 codes C18.0, C18.2, C18.3, C18.4, C18.5, C18.6, C18.7, C18.8, C18.9, C19 and C20 were defined as CRC cases. Self-reported cases of cancers of bowel, colon or rectum, if not confirmed by the ICD9 or ICD10 codes were excluded from the analysis. Healthy control individuals without history of cancer and/or colorectal adenoma were included in the analysis after matching 1 case to 4 controls by age, gender, date of blood draw, ethnicity and region of residence (two first letters of postal code). Individuals were excluded with: history of cancer including non-melanoma cancers (n=3,085), discordant sex information (n = 17) and sex chromosome aneuploidy (n=15), duplication or first-degree relatedness (n = 228), self-defined non-white individuals (n=611), evidence of non-white European ancestry using PCA in conjunction with 1000G samples (n = 74; cut-off based on visual assessment of the top two principal components of the controls), abnormal heterozygosity (n=29). In total, 3,301 cases and 11,382 controls were included in the analysis.

***Croatia CRC case-control study***

This case-control study consists of 764 CRC cases and 460 population based controls. Detailed description of the participants is presented elsewhere. Genotyping of cases and controls was conducted using OmniExpressExome BeadChip 8v1.1 or 8v1.4 (Illumina Inc., San Diego, CA). Individuals were excluded with: high missing rate (>0.05%, n=56), discordant sex information (n = 9), duplication or first-degree relatedness (n = 5), evidence of non-white European ancestry using PCA in conjunction with 1000G samples (n = 12; cut-off based on visual assessment of the top two principal components of the controls), abnormal heterozygosity (n=2). In total, 689 cases and 441 controls were included in the analysis.

**Imputation and genetic analysis**

Details of imputation and related quality control procedures have been described previously. Prior to imputation SOCCS/GS and Croatia case-control studies were phased using SHAPEIT (v2.r837) software. Imputation of untyped genotypes was performed using IMPUTEv2 software. The reference panels used were the UK10k release (ALSPAC and TWINSUK studies, Apr 2014 release), and the 1000 Genome V3 (Dec 2013 release). We excluded variants that have discrepancies in strand and position information across two reference panels (<1% of variants, see additional information). The two reference panels were merged in IMPUTE2 using the -merge-ref-panel option and imputed in 5Mbp chunk. Monomorphic variants, rare variants with allele counts <20 and poorly imputed SNPs (*i.e.* INFO score of <0.8) were excluded from the analysis. LBC was imputed to 1000 Genome version 3 (March 2012 release) using minimac.UK Biobank case-control study was imputed using the Haplotype Reference Consortium (HRC) panel. Detail of imputation and sample processing are described elsewhere (see additional information).

The association between each genetic variant and the risk of CRC was assessed in SNPTEST (v2.5.1) by a frequentist association test under an additive model and taking imputation uncertainty into account.

Information on imputation quality of imputed variants and/or genotyping status for the vitamin D variants used as instrumental variables are presented in the supplementary table S6.

Table S1 Summary of the eleven genome-wide association studies of colorectal cancer (18,967 CRC cases and 48,158 controls) for summary statistics MR.

| **Series** | **Study setting** | **Study centre** | **Sampling** | **No. cases** | **No. controls** |
| --- | --- | --- | --- | --- | --- |
| CCFR1 | Colon Cancer Family Registry | University of Southern California | Recently diagnosed cases reported to population complete cancer registries in the USA (Seattle Familial Colorectal Cancer Registry). Canada (Ontario Familial Cancer Registry) and Australia (Australasian Colorectal Cancer Family Study). Population-based controls | 1,290 | 1,055 |
| CCFR2 | Colon Cancer Family Registry | University of Southern California | Recently diagnosed cases reported to population-based cancer registries in the USA (Seattle Familial Colorectal Cancer Registry, Mayo Clinic Cooperative Family Registry for Colon Cancer Studies, USC Consortium Colorectal Cancer Family Registry, University of Hawaii Colorectal Cancer Family Registry). Canada (Ontario Familial Cancer Registry), Australia (Australasian Colorectal Cancer Family Study). Unaffected family controls. | 796 | 2,236 |
| COIN | COIN trial | Cardiff University | Multicentre study of cetuximab and other therapies in metastatic CRC. Cases recruited as a clinical-based series and controls as population-based series. Controls were unselected blood donors | 2,244 | 2,162 |
| FINLAND | Finnish Colorectal Cancer Predisposition Study | Helsinki University | Cases requited through Finnish Hospitals and Finnish Cancer Registry. Population-based controls from FINRISK, Health 2000, Finnish Twin Cohort and Helsinki Birth Cohort Studies | 1,172 | 8,266 |
| UK1 | CORGI (Colorectal Tumour Gene Identification Consortium) | Oxford University | Cases enriched for family history of CRC, ascertained through UK clinical genetics clinics. Spouse controls with no personal history or family history of CRC. | 940 | 965 |
| Scotland1 | COGS (Colorectal Cancer Susceptibility Study) | Edinburgh University | Population-based incidence cases aged <55 at diagnosis; Scotland. Population-based controls frequency matched by area of residence within Scotland | 1,012 | 1,012 |
| VQ58 | Cases: VICTOR, post treatment stager of a phase III, randomised trial of rofecoxib (VIOXX) in patients after potentially curative therapy. QUASAR2, multi-centre study of capectibine±bevacizumb as adjuvant treatment. 1958 Birth cohort controls | Oxford University | Cases recruited as a clinical-based series and controls as population-based series | 1,800 | 2,690 |
| Scotland SOCCS3/Generation Scotland | Cases: Scottish colorectal cancer study3; controls: Generation Scotland population based controls and SOCCS study controls | Edinburgh University | Population based incidence cases and population based controls from Scotland | 4,772 | 12,158 |
| Scotland SOCCS/LBC | Cases: SOCCS  Controls: the Lothian Birth Cohorts 1921 and 1936 | Edinburgh University | Population based incidence cases and population based controls from Scotland | 494 | 1522 |
| Croatia case-control study | Cases：hospital based  Controls: ‘10,001 Dalmatians’ study | [University Hospital Centre Zagreb](https://en.wikipedia.org/wiki/University_Hospital_Centre_Zagreb) | Colorectal cancer cases were recruited at departments of surgery in two hospitals in Zagreb, Croatia. Controls were selected from the genetic study ‘10,001 Dalmatians’ in Croatia | 764 | 460 |
| UK Biobank | Cases: prevalence (~51%) and incidence cases of colorectal cancer across UK: controls: population based controls without history of cancer | Edinburgh University | Population based incidence and prevalence cases of colorectal cancer,UK. Population based controls without history of cancer and/or colorectal adenoma matched 1 to 4  by age, gender ,date of blood draw, ethnicity and region of residence (two first letters of postal  code) | 3,683 | 15,642 |
| Combined |  |  |  | 18,967 | 48,168 |

MR, Mendelian randomisation; SOCCS, study of colorectal cancer in Scotland.

Table S2 Results of tests on correlation between GRS and common confounders in healthy controls

|  | **P-value for correlation significance test*** | |
| --- | --- | --- |
| **Dataset** | UKB | SOCCS |
| **Confounders** |  |  |
| Height | 0.875 | 0.228 |
| Weight | 0.428 | 0.908 |
| BMI | 0.228 | 0.941 |
| Age | 0.982 | 0.419 |
| Gender | 0.062 | 0.243 |
| Smoking status | 0.474 | NA |
| Alcohol intake | 0.19 | NA |
| Physical activity | 0.314 | NA |
| Assessment/Recruitment centre | 0.156 | 0.154 |

*Significance of Spearman’s correlation coefficient between genetic risk score and continuous variables was tested, and analysis of variance (ANOVA) was applied for categorical variables.

GRS, weighed genetic risk score; UKB, UK biobank; SOCCS, Study of Colorectal Cancer in Scotland; NA, not available.

Table S3 Power calculation for individual and summary level MR across various effect sizes and proportions of vitamin D variance explained by instrumental variable

|  | | **Range of effects (Odds Ratios) on CRC risk per 1 SD of increased vitamin D risk*** | | | | | | |
| --- | --- | --- | --- | --- | --- | --- | --- | --- |
| **Proportion of variance in 25-OHD explained by the IV** | | **0.98** | **0.95** | **0.9** | **0.828** | **0.8** | **0.7** | **0.6** |
|  | Individual level MR (n=32,788, proportion of cases=0.30, α=0.05) | | | | | | | |
| **1%** | | 5% | 7% | 14% | 33% | 43% | 79% | 97% |
| **2%** | | 6% | 9% | 23% | 57% | 71% | 98% | 100% |
| **2.84%** | | 6% | 11% | 30% | **72%** | 85% | 100% | 100% |
| **5%** | | 7% | 16% | 48% | 92% | 98% | 100% | 100% |
|  | Summary level MR (n=57,811, proportion of cases=0.31, α=0.05) | | | | | | | |
| **1%** | | 6% | 9% | 21% | 52% | 66% | 96% | 100% |
| **2%** | | 6% | 13% | 37% | 81% | 92% | 100% | 100% |
| **2.84%** | | 7% | 16% | 49% | **93%** | 98% | 100% | 100% |
| **5%** | | 8% | 26% | 73% | 99% | 100% | 100% | 100% |

*Power was calculated for the range of effect sizes from OR-0.6 to OR=0.98 with the effects of one standard deviation of 25-OHD level on CRC risk in the SOCCS case-control study = 0.828

MR, Mendelian Randomisation; IV, instrumental variable; CRC, colorectal cancer; SD, standard deviation.

Table S4 Results of MR analyses using different combinations of the instrumental genetic variants

| **IV Combination** | **Two-SNP IV** | | **Three-SNP IV** | | **Four-SNP IV** | | **Five-SNP IV** | |
| --- | --- | --- | --- | --- | --- | --- | --- | --- |
| **MR Methods** | **OR (95% CI)** | **P** | **OR (95% CI)** | **P** | **OR (95% CI)** | **P** | **OR (95% CI)** | **P** |
| IVW | 1.14(0.62-2.10) | 0.791 | 1.23(0.71-2.13) | 0.531 | 1.17(0.69-1.96) | 0.550 | 1.09(0.66-1.81) | 0.730 |
| Simple median | NA |  | 1.68(0.79-3.58) | 0.179 | 1.13(0.59-2.19) | 0.709 | 0.76(0.36-1.63) | 0.49 |
| Weighted median | NA |  | 1.36(0.70-2.64) | 0.363 | 1.22(0.65-2.28) | 0.537 | 1.14(0.62-2.13) | 0.68 |

IV, instrumental variable; MR, Mendelian randomisation; SNP, single nucleotide polymorphism; IVW, inverse variance weighted, NA, not available;

Two-SNP IV: rs10741657,rs12785878; Three-SNP IV: rs10741657,rs12785878,rs17216707; Four-SNP IV: rs10741657,rs12785878,rs17216707,rs10745742;

Five-SNP IV: rs10741657,rs12785878,rs17216707,rs10745742,rs8018720.

Table S5 Results of stratified MR analyses based on anatomical location

|  | **Causal estimate(Odds ratio)*** | | | | | | |
| --- | --- | --- | --- | --- | --- | --- | --- |
|  | UKB | SP1 | SOCCS | Croatia | Overall estimate | P-value | P-het |
| Proximal colon | 0.33(0.08-1.27) | 6.06(0.39-93.81) | 1.61(0.56-4.65) | 2.42(0.04-149.19) | 1.16(0.35-3.84) | 0.805 | 0.169 |
| Distal colon | 0.73(0.26-2.06) | 0.33(0.03-3.90) | 0.70(0.24-2.05) | 18.82(0.36-973.41) | 0.74(0.37-1.50) | 0.410 | 0.390 |
| Rectum | 1.01(0.31-3.29) | 4.13(0.39-43.91) | 0.39(0.13-1.12) | 19.26(0.47-785.08) | 1.21(0.34-4.29) | 0.765 | 0.089 |

*causal effect was estimated using weighted genetic score under a univariable model;

P_het_, P values of Chi-square Q test for heterogeneity

Table S6 Genotyping status and imputation quality metrics (info statistics) for the imputed genetic variants used as instrumental variables

| ID |  | CCFR1 | CCFR2 | COIN | FINLAND | UK1 | Scotland 1 | VQ58 | Scotland SOCCS3/Generation Scotland | Scotland SOCCS/LBC | Croatia case-control study | UK Biobank |
| --- | --- | --- | --- | --- | --- | --- | --- | --- | --- | --- | --- | --- |
| rs10741657 | 11 | 0.99 | 0.99 | 0.96 | 0.99 | 0.99 | 0.99 | 0.999 | genotyped | 0.99 | genotyped | genotyped |
| rs10745742 | 12 | 0.99 | 0.99 | 0.98 | 0.99 | 0.99 | 0.99 | 0.99 | 0.99 | 0.99 | 0.99 | 0.99 |
| **rs12785878** | 11 | 0.99 | 0.99 | 0.92 | 0.99 | 0.99 | 0.99 | 0.99 | 0.99 | 0.98 | 0.99 | 1 |
| **rs17216707** | 20 | genotyped | 0.92 | 1 | 0.95 | 0.95 | 0.96 | 0.92 | 0.93 | 0.93 | 0.93 | 0.97 |
| **rs3755967** | 4 | genotyped | 0.99 | 0.98 | 0.99 | 0.99 | 0.99 | 0.98 | 0.99 | 0.96 | 0.99 | 0.99 |
| rs8018720 | 14 | 0.99 | 0.99 | 0.99 | 0.99 | 0.99 | 0.99 | 0.99 | 1 | 0.99 | genotyped | genotyped |

**References**

1. Study C, Houlston RS, Webb E, Broderick P, Pittman AM, Di Bernardo MC et al. Meta-analysis of genome-wide association data identifies four new susceptibility loci for colorectal cancer. Nature genetics 2008; 40:1426-35.

2. Houlston RS, Cheadle J, Dobbins SE, Tenesa A, Jones AM, Howarth K et al. Meta-analysis of three genome-wide association studies identifies susceptibility loci for colorectal cancer at 1q41, 3q26.2, 12q13.13 and 20q13.33. Nature genetics 2010; 42:973-7.

3. Theodoratou E, Farrington SM, Tenesa A, McNeill G, Cetnarskyj R, Barnetson RA et al. Dietary vitamin B6 intake and the risk of colorectal cancer. Cancer epidemiology, biomarkers & prevention : a publication of the American Association for Cancer Research, cosponsored by the American Society of Preventive Oncology 2008; 17:171-82.

4. Deary IJ, Gow AJ, Pattie A, Starr JM. Cohort profile: the Lothian Birth Cohorts of 1921 and 1936. International journal of epidemiology 2012; 41:1576-84.

5. Smith BH, Campbell A, Linksted P, Fitzpatrick B, Jackson C, Kerr SM et al. Cohort Profile: Generation Scotland: Scottish Family Health Study (GS:SFHS). The study, its participants and their potential for genetic research on health and illness. International journal of epidemiology 2013; 42:689-700.

6. Timofeeva MN, Kinnersley B, Farrington SM, Whiffin N, Palles C, Svinti V et al. Recurrent Coding Sequence Variation Explains Only A Small Fraction of the Genetic Architecture of Colorectal Cancer. Scientific reports 2015; 5:16286.

7. Davies G, Tenesa A, Payton A, Yang J, Harris SE, Liewald D et al. Genome-wide association studies establish that human intelligence is highly heritable and polygenic. Molecular psychiatry 2011; 16:996-1005.

8. Zgaga L, Theodoratou E, Farrington SM, Din FV, Ooi LY, Glodzik D et al. Plasma vitamin D concentration influences survival outcome after a diagnosis of colorectal cancer. Journal of clinical oncology : official journal of the American Society of Clinical Oncology 2014; 32:2430-9.

9. Wain LV, Shrine N, Miller S, Jackson VE, Ntalla I, Soler Artigas M et al. Novel insights into the genetics of smoking behaviour, lung function, and chronic obstructive pulmonary disease (UK BiLEVE): a genetic association study in UK Biobank. The Lancet Respiratory medicine 2015; 3:769-81.

10. Kirac I, Matosevic P, Augustin G, Simunovic I, Hostic V, Zupancic S et al. SMAD7 variant rs4939827 is associated with colorectal cancer risk in Croatian population. PloS one 2013; 8:e74042.

11. Orlando G, Law PJ, Palin K, Tuupanen S, Gylfe A, Hanninen UA et al. Variation at 2q35 (PNKD and TMBIM1) influences colorectal cancer risk and identifies a pleiotropic effect with inflammatory bowel disease. Human molecular genetics 2016; 25:2349-59.

12. Rodriguez-Broadbent H, Law PJ, Sud A, Palin K, Tuupanen S, Gylfe A et al. Mendelian randomisation implicates hyperlipidaemia as a risk factor for colorectal cancer. International journal of cancer 2017; 140:2701-8.

13. Delaneau O, Marchini J, Zagury JF. A linear complexity phasing method for thousands of genomes. Nat Methods 2011; 9:179-81.

14. Howie BN, Donnelly P, Marchini J. A flexible and accurate genotype imputation method for the next generation of genome-wide association studies. PLoS Genet 2009; 5:e1000529.

15. McCarthy S, Das S, Kretzschmar W, Delaneau O, Wood AR, Teumer A et al. A reference panel of 64,976 haplotypes for genotype imputation. Nature genetics 2016; 48:1279-83.

16. Marchini J, Howie B, Myers S, McVean G, Donnelly P. A new multipoint method for genome-wide association studies by imputation of genotypes. Nature genetics 2007; 39:906-13.
